# Role of the SOX family in cancer immune evasion: Emerging player and promising therapeutic opportunities

**DOI:** 10.1097/MD.0000000000041393

**Published:** 2025-01-31

**Authors:** Jinke Li, Yawen Xu, Yunying Han, Aifu Yang, Miaoshan Qian, Bo Wang

**Affiliations:** a Department of General Surgery, Longnan First People’s Hospital, Longnan, China.

**Keywords:** cancer, immune evasion, SOX family

## Abstract

Cancer immune evasion is one of the important mechanisms for cancer development, which is essential to developing novel immunotherapeutic strategies. The SOX (SRY-related HMG-box) family of transcription factors plays a crucial role in normal physiology as well as in a variety of human diseases especially cancer. It has been shown that SOX is involved in cancer immune evasion processes. This mini-review aimed to summarize how SOX family members induce cancer immune evasion by regulating antigen presentation, shaping the tumor immunosuppressive milieu, and controlling regulatory immune checkpoint inhibitors like programmed death ligand 1. Thorough exploration of SOX family will help uncover the mechanism of cancer immune evasion, and provide new ideas and targets for the development of immunotherapy strategies.

## 1. Introduction

Cancer immune evasion refers to various ways in which cancer cells evade host immune surveillance and elimination. These strategies include alterations in antigen presentation pathways,^[1]^ induction of an immune-suppressive microenvironment,^[2]^ as well as activation of immune checkpoint signals.^[3]^ Immune evasion not only drives tumor growth but also results in resistance of tumors against existing immune-based therapies. Thus, understanding molecular mechanisms underlying cancer immune evasion is crucial for development of new anticancer therapies.

The SRY-related HMG-box (SOX) family member of transcription factors is a highly conserved group that plays important roles in embryonic development, cell differentiation, and maintenance of tissue specificity.^[4–10]^ Besides their roles in developmental biology, recent studies have shown that several members of the SOX family including SOX2, SOX4, SOX9, etc are abnormally expressed in various types of tumors and closely associated with tumorigenesis and progression.^[9,11–15]^

Numerous pathways have indicated that SOX family members are involved in immunity evasion including modulation of tumor cells’ antigen presentation abilities alteration of immune suppressive cellular activities and regulation of immune checkpoint molecules present on cancer cells’ surfaces. For example, overexpression of SOX2 correlates with upregulation of programmed cell death ligand 1 (PD-L1) levels on tumor cell surfaces which promotes immune escape by tumor cells.^[16]^ In addition, SOX2 has been found to enhance immune suppressive environment further assisting tumor cells to escape immune surveillance by potentiating recruitment and activation of regulatory T cells (Tregs).^[17]^ This mini-review will overview the structure and function of SOX family, its roles in cancer immune evasion and potential targets for therapy.

## 2. Structure and biological functions of the SOX family

**Figure 1.**
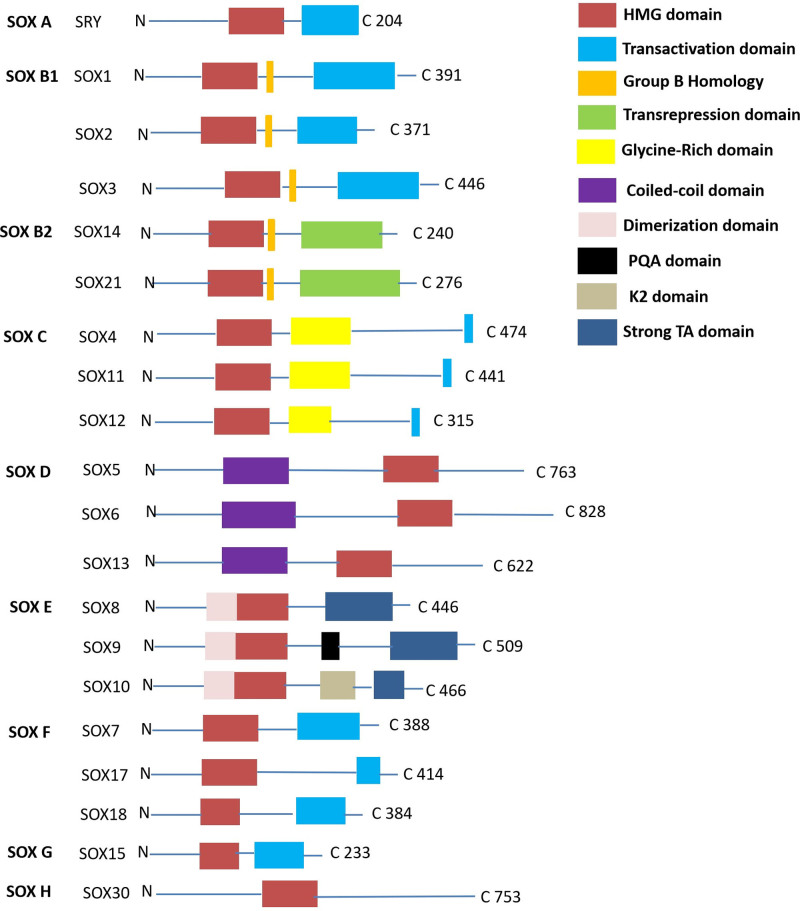
The structural organization of SOX transcription factors. The SOX transcription factor family consists of 9 subgroups, each containing a highly conserved HMG domain consisting of 79 amino acids. The structure and function of members within the same subgroup are similar. This diagram shows several domains, including the high mobility group (HMG) domain (reddish brown box), transactivation domain (blue box), group B homology (orange yellow box), transrepression domain (green box), glycine-rich domain (yellow box), coiled-coil domain (purple box), dimerization domain (pink box), PQA domain (black box), K2 domain (brown box), and strong TA domain (deep blue box).

Based on their sequence similarity and functional properties, members of the SOX family can be classified into 8 main groups: B, C, D, E, F, G, H, and I. The members of each group have analogous DNA-binding domains and functional characteristics.^[18]^ SOX proteins contain a conserved core structure characterized by a high mobility group box domain composed of about 79 amino acids that interacts with the minor groove of DNA.^[19]^ This interaction causes DNA bending, which alters chromatin organization to modulate gene transcriptional activity. Some SOX proteins also have different functional domains other than high mobility group box such as a transcriptional activation or repression domain.^[20]^ These functional domains can interact with other proteins to enhance or repress transcriptional regulation.^[21–23]^ SOX family genes are highly conserved in multicellular organisms indicating that they play important roles in biological development.^[24,25]^ In humans and other animals, abnormal expression of SOX genes is closely associated with various diseases including cancer.^[26–31]^ This means that understanding the structural features of these genes is crucial for examining their role in immune evasion. The structural organization of SOX transcription factors was shown in Figure 1.

The SOX family is involved in multiple biological processes, including cell development, differentiation, and may lead to disease. Findings from Hu show that SOX2 as one of the core molecules maintains embryonic stem cells and neural precursor cells in an undifferentiated state.^[32]^ It has shown that SOX9 is crucial for chondrocyte differentiation and its defect may result in various skeletal malformations such as nutritional rickets and sex reversal.^[33]^ In addition, it has been found that SOX17 plays a crucial role in the formation of tissues derived from the endoderm including liver and pancreas.^[34]^ Besides, SOX genes are also involved in the maintenance of certain cell type function. For instance, SOX10 acts as an important factor in the development of neural crest and plays a role in the regulation of the peripheral nervous system and it is important for maintaining the function of melanocytes and some neuronal cells in adults.^[35]^ The functional role of SOX family proteins was summarized in Table 1.

**Table 1:** The functional role of SOX family proteins in cancer.

Group	Gene	Physiological function	Role in cancer	References
A	Sry	Mammalian testis-determining gene.	SRY promotes the migration and invasion of hepatoma cells.	[36,37]
B1	Sox1	SOX1 is involved in the regulation of neural stem cell differentiation.	SOX1 inhibits the progression of hepatocellular carcinoma, cervical cancer, nasopharyngeal carcinoma and lung cancer.	[38–42]
	Sox2	SOX2 plays an important role in the pluripotency and self-renewal of embryonic stem cells.	SOX2 induces immune evasion of CD8+ T-cell killing by alleviating the JAK–STAT pathway and interferon-stimulated gene resistance signature expression.	[43,44]
	Sox3	SOX3 is very important for gonadal function.	SOX3 promotes the progression of gastric cancer and osteosarcoma.	[45]
	Sox19	SOX19 regulates ovarian steroid production and forebrain development.	Unknown.	[46,47]
B2	SoxB2.1	SoxB2.1, also known as dichaete, is expressed in local interneurons and plays a role in the development of olfactory circuits.	Unknown.	[48]
	Sox14	Sox14 regulates neurogenesis.	Sox14 promotes the proliferation and invasion of cervical cancer.	[49]
	Sox21	Sox21 is involved in neural stem cell differentiation.	SOX21 promotes the progression of colon cancer, pancreatic cancer, cervical cancer and nephroblastoma.	[50–54]
C	Sox4	Sox4 induces lymphocyte differentiation, neurogenesis, cardiovascular development,cartilage growth plate formation.	SOX4 inhibits the expression of genes in innate and adaptive immune pathways that are critical to protective tumor immunity.	[55–57,88]
	Sox11	SOX11 is involved in neurogenesis and cartilage growth plate formation.	SOX11 expression is associated with the immunosuppressive microenvironment characterized by increased Treg cell infiltration and down-regulation of antigen processing, and presentation, and T cell activation.	[57–59]
	Sox12	SOX12 regulates lymphocyte differentiation.	SOX12 increases intratumoral regulatory T-cell (Treg) infiltration and decreases CD8+ T-cell infiltration in liver cancer.	[60,61]
	Sox22	SOX22 is expressed in a variety of fetal and adult organs and tissues.	Unknown.	[62]
	Sox24	Sox24 regulates oogenesis.	Unknown.	[63]
D	Sox5	SOX5 activates neural crest specification.	SOX5 promotes tumor progression and immune evasion in triple-negative breast cancer through transcriptional activation of circ_0084653.	[64,65]
	Sox6	SOX6 promotes survival and maturation of erythroid cells.	SOX6 protein has multiple helper epitopes, which can induce anti-tumor activity in glioma.	[66,67]
	Sox13	SOX13 regulates lymphocyte differentiation.	SOX13 decreased CD8+ T cell activity in breast cancer.	[68,69]
	Sox23	SOX23 is expressed in embryonic ovary and brain.	Unknown.	[70]
E	Sox8	SOX8 is involved in testis differentiation and osteogenesis.	SOX8 promotes proliferation of tongue squamous cell carcinoma.	[36,71,72]
	Sox9	SOX9 promotes the development of testicular and bone tissue, promotes the layering of the trunk neural crest, and maintains the homeostasis of stem cells in the tissue.	SOX9 helps tumor cells maintain a stem-like state and evade innate immunity by remaining dormant for extended periods.	[73–76]
	Sox10	SOX10 regulates peripheral nerve development.	SOX10 regulates immune checkpoint protein expression and anti-tumor immunity in melanoma.	[77,78]
F	Sox7	SOX7 regulates hematopoietic development and lymphatic endothelial cell growth.	SOX7 inhibits the proliferation of glioma, renal cell carcinoma, colorectal cancer and acute leukemia.	[79–83]
	Sox17	SOX17 regulates the development of cardiovascular system.	SOX17 inhibits tumor cells’ ability to sense and respond to IFNγ, thereby preventing anti-tumor T cell responses.	[84,85]
	Sox18	SOX18 regulates vascular and lymphatic development.	SOX18 promotes the accumulation of Tregs and immunosuppressive tumor-associated macrophages (TAMs) in the liver cancer microenvironment by transactivating PD-L1 and CXCL12.	[86–88]
G	Sox20	SOX20 is expressed in fetal testis, but its physiological function is unknown.	Unknown.	[89–91]
	Sox15	SOX15 regulates stem cell pluripotency and determines neural fate during differentiation.	SOX15 inhibits the proliferation of glioma and prostate cancer.	[92–94]
H	Sox30	SOX30 is a key regulator of germ cell meiosis and testis differentiation.	SOX30 inhibits invasion and metastasis of lung cancer, prostate cancer, and acute myeloid leukemia. SOX30 is involved in immune infiltration, providing an important bridge between tumor and immunity.	[95–97]
I	Sox31	Sox31 is involved in anterior–posterior regionalization of the central nervous system.	Unknown.	[98]

SOX family genes are not only involved in normal biological processes but also play crucial roles in the development of various diseases especially in human tumors. For example, the expression of SOX2 is up-regulated in multiple cancers regulating diverse biological functions such as self-renewal capabilities of tumor cells, proliferation rates, chemotherapeutic resistance, etc.^[99–101]^ Moreover, it was found that abnormal expressions of SOX could mediate immune evasion by cancer cells. This will be elaborated on in detail in Section 3.

## 3. The role of the SOX family in tumor immune evasion

### 3.1. Activation and recruitment of immunosuppressive cells

The SOX family of transcription factors plays an important role in tumor immune evasion, particularly through modulating the activation and recruitment of immune-inhibitory cells. These cells include Tregs, tumor-associated macrophages (TAMs) and myeloid-derived suppressor cells, which are central to impeding antitumor immune response.^[102,103]^ Members of the SOX family such as SOX2 and SOX9 regulate the function and number of these cells through various mechanisms, hence affecting tumor immune microenvironment.

One of the known members of the SOX family of transcription factors that impact on the immune suppressive environment is SOX2. In some tumors, SOX2 expression is associated with increased Tregs, which might occur by regulating immunosuppressive cytokines.^[104]^ In addition, SOX2 facilitates lung metastasis in breast cancer by recruiting TAMs.^[17]^ Chen et al found that SOX9 is a key regulatory molecule that recruits tumor-associated macrophages and inhibits the expression of CD8+ T cells and promotes pancreatic cancer progression.^[105]^

The SOX family of transcription factors enhances tumor immune evasion capacity through activation and recruitment of immune suppressor cells. These genes change the tumor microenvironment by regulating cell-cell interactions within tumor cells and immune cells, thereby promoting immune evasion. Therefore, studying the role of SOX family in modulating immune suppressor cells may not only deepen our understanding of the mechanism underlying tumor immune evasion, but also provide new therapeutic targets for designing novel immune-therapy strategies.

### 3.2. Regulation of antigen presentation

Antigen presentation is a critical step in tumor immune surveillance, which involves expression of major histocompatibility complex (MHC) molecules on the surface of tumor cells exposing T-cells to tumor-specific antigens.^[106]^ SOX family members act on regulating antigen presentation so as to affect visibility of tumor cells to immune system.

SOX2 is one of the increased expressed SOX family members in many tumors, and it is also regulated in antigen presentation through its influence on MHC class I molecules. A study showed that high expression of SOX4 presented low expression of MHC class I in the of tumor microenvironment of cervical cancer.^[107]^ In colorectal cancer, the expression of SOX17 is also linked to a diminished antigen presentation. SOX17 reduces antitumor T-cell responses by decreasing MHC-I expression, thereby facilitating tumor cell immune evasion and promoting colon cancer initiation and progression.^[84]^ In the context of tumor immune evasion, up-regulation of SOX4 is not only related to cell proliferation and survival but also affects antigen presentation by tumor cells. Upon the promotion of immune evasion, SOX4 induced expression of MHC class I inhibited protective tumor immunity in triple-negative breast cancer.^[55]^

The SOX family of transcription factors plays a critical role in affecting tumor immune evasion by controlling the expression of antigen presentation associated molecules. The regulatory activities of these genes attenuate immune visibility of tumor cells thereby allowing them to survive and proliferate under host immune surveillance. This discovery underscores the importance of SOX family members as candidate targets for cancer therapy, especially in the development of treatment strategies aimed at enhancing anti-tumor immune responses. Further studies on the role of SOX family members in regulating antigen presentation could lead to development of novel therapeutic approaches that may enhance immune system mediated killing of tumors by restoring or enhancing tumor cell antigen presenting capacity.

### 3.3. Immune checkpoint regulation

In the complex mechanism that sustains tumor immune evasion, immune checkpoint molecules play a central role. Through binding with particular immune cell surface receptors, their activation is suppressed leading to evasion of tumor cells from immune system attack.^[108]^ The most common immune checkpoint molecules involve PD-L1 and progammed cell death protein1 (PD-1). In recent years, it has been found out that SOX family transcription factors play an important role in controlling these immune checkpoint molecules which has major implications on the development of cancer treatment based on immunotherapy.^[44,55,60,68,84,86,109,110]^

One of the SOX family members that are up-regulated in expression in multiple cancers is SOX2, and it has been shown to play an important role in regulating PD-L1 expression. For example, studies show that SOX2 can increase PD-L1 expression in stem-like cells of glioblastoma, resulting in tumor immune escape.^[16]^ Research indicates that Janus kinase–signal transducer and activator of transcription pathways controls expression of a wide range of immune checkpoint proteins, such as galectin-9, PD-1, TIM-3, PD-L1, and indoleamine 2,3-dioxygenase 1.^[111–113]^ Furthermore, SOX2 can affect the Janus kinase–signal transducer and activator of transcription pathway within the tumor microenvironment therefore affecting expression of PD-L1 and T-cell-mediated cytotoxicity in melanoma cells.^[44]^ An important role that SOX4 plays in the advancement of cancer should not be underestimated as far as the regulation of immune checkpoints is concerned. Research has shown that anti-PD-1 resistance and immune evasion enhanced by SOX4 in non-small cell lung cancer.^[109]^ Importantly, SOX4 can redirect TGF-β-mediated SMAD3-transcriptional output in a context-dependent manner to promote tumorigenesis.^[114]^ This pathway regulates expression of a number of key immune checkpoint proteins including PD-L1, VISTA, galectin-9^[115]^ as well as indoleamine-2,3-dioxygenase.^[116]^ This cross-linking between SOX4 and TGF-β-Smad3 signaling pathways plays a crucial role in the expression of immune checkpoint proteins. Another SOX family member, SOX6, promotes PD-L1 levels in cervical cancer.^[117]^ Specifically, miR-18a increases PD-L1 levels by targeting SOX6 to activate the Wnt/beta-catenin pathway and inactivate p53 signaling. In addition, a study from Dong et al demonstrated that SOX10 plays a crucial role in regulating expression of immunotherapy checkpoint proteins and antitumor immunity in melanoma.^[77]^ They found the transcription levels of the immune checkpoint proteins HVEM and CEACAM1 in the SOX10 knockout cells were significantly changed.

So far, it is evident that SOX family members play crucial roles in cancer immunoescape especially by regulating immune checkpoint molecules such as PD-L1 through several mechanisms. Therefore, these findings not only enlighten us on the complexity of regulation in tumor immunity but also offer opportunities for devising new immunotherapic strategies. Targeting SOX family genes for therapy like with inhibitors of SOX2, SOX4, SOX6, SOX10, SOX12, or SOX18 might represent novel hopes for treatment because they could restore normal immune system cytotoxic function via downregulation of PD-1 expression in SOX family-gene therapies.^[44,60,86,109,117–120]^

## 4. Potential of SOX family genes as therapeutic targets

The SOX gene family has become a potential therapeutic target in the study of the cellular immune escape mechanism of tumor for its important roles in regulating proliferation and immune regulation of cancer cell. As a better understanding of the role of this family in tumor development emerges, the therapeutics strategy to block SOX gene is gradually revealing its therapeutic potential.

One of the most direct therapeutic strategies towards SOX family genes is small molecule inhibitors. For instance, small molecule inhibitors against SOX18 have been shown to disrupt the cell cycle and inhibit cell growth.^[121]^ These inhibitors act on the function of SOX18 in tumor cells by either inhibiting its DNA binding ability or its transcriptional activity.^[122]^ Similarly, inhibitors against SOX family are also being developed with the aim of inhibiting their role in tumor progression and immune evasion in the future.

Targeting the mRNA of the SOX family of genes using RNA interference technologies such as siRNA and shRNA is a potential strategy that might reduce their expression in tumor cells. Consequently, monoclonal antibodies directed against SOX proteins can also be developed as therapeutic tools for tumors, especially in those cancer types where SOX gene expression is closely associated with disease progression.

The SOX gene family plays a crucial role in tumor growth regulation, immune evasion as well as the tumor microenvironment. Therefore, these genes provide new pathways of treating cancers. It is expected that more therapy approaches will be developed based on SOX genes in the future bringing hope to cancer patients.

## 5. Conclusion

Tumor immune evasion is a critical factor in the survival and progression of tumors involving various cell types and molecular mechanisms. The SOX family of transcription factors play multiple roles in this process, ranging from regulating antigen presentation by tumor cells and expressing immune checkpoints on them to activating and recruiting immunosuppressive cells. In these ways, SOX genes directly and indirectly influence the tumor immune microenvironment, facilitating immune escape by tumor cells from immune system surveillance.

Research has shown that the Sox family members such as SOX2, SOX4, and SOX9 play critical roles in immune evasion of tumors.^[44,109,118,123]^ They do these functions by either regulating the expression of immune checkpoint molecules, like PD-L1, which affect the tumor cells’ antigen presentation capabilities or causing changes in the tumor microenvironment via activation of immunosuppressive cells such as Tregs, myeloid-derived suppressor cells, and TAMs.^[44,109,118,123]^ These mechanisms make SOX family potential and important targets for cancer therapy.^[36–43,45–54,56–59,61–67,69–76,78–83,85,87–98,124]^ Following the development of these avenues of research, future advancements in tumor immunotherapy could be facilitated, which would result in the provision of more efficient treatment options to patients.

## Author contributions

**Conceptualization:** Jinke Li, Yunying Han, Bo Wang.

**Formal analysis:** Yawen Xu, Miaoshan Qian, Bo Wang.

**Funding acquisition:** Bo Wang.

**Investigation:** Yawen Xu, Miaoshan Qian, Bo Wang.

**Methodology:** Yunying Han, Aifu Yang.

**Project administration:** Yawen Xu.

**Resources:** Aifu Yang, Miaoshan Qian.

**Software:** Jinke Li, Yunying Han.

**Supervision:** Bo Wang.

**Validation:** Yunying Han.

**Writing – original draft:** Jinke Li.

**Writing – review & editing:** Bo Wang.
